# Relationship of BMI with the diet, physical activity and oral hygiene practices amongst the dental students

**DOI:** 10.1186/s12903-022-02318-8

**Published:** 2022-07-28

**Authors:** Beenish Fatima Alam, Nabeela Abbasi, Talib Hussain, Malik Arshman Khan, Muhammad Aamir Ghafoor Chaudhary, Faiza Ijaz

**Affiliations:** 1 Department of Oral Biology, Bahria University Dental College, Karachi, Pakistan; 2 Department of Oral Biology, Rawal Institute of Health Sciences, Islamabad, Pakistan; 3 Department of Oral Biology, Women Medical and Dental College, Abbottabad, Pakistan; 4 Department of Oral Biology, Abbottabad International Dental College, Abbottabad, Pakistan; 5 Department of Prosthodontics, Islamic International Dental College, Riphah International University, Islamabad, Pakistan; 6 Department of Oral Biology, Women Medical and Dental College, Abbottabad, Pakistan

**Keywords:** BMI, Obese, Dental students, Tooth brushing, Physical activity

## Abstract

**Background:**

Regardless of attaining adequate knowledge regarding oral hygiene, physical activity, and healthy eating habits, dental students still face oral health problems. This study was aimed to assess the association of oral hygiene habits, physical activity, and eating habits with the BMI in the dental students.

**Method:**

This multi centric cross-sectional study was conducted from January to May 2021 in Pakistan. Three hundred and eighty-six study participants enrolled as undergraduate dental students, both males and females, were included in the study. A questionnaire used to gather data, was modified from a study conducted by Jouhar et al. Chi-square testing was used in order to assess the relationship between two categorical variables. Linear regression was performed to assess the association with putative confounders. Statistical significance was considered for p value < 0.05.

**Results:**

Regarding brushing teeth, 57% of the underweight individuals brushed once daily, 69.8% of the healthy, 79.2% of overweight, and 48% of obese participants brushed twice. Horizontal brushing technique was performed by 50% of the underweight participants, followed by scrub technique. A soft bristled brush was frequently used by underweight (42.9%) and healthy (66%) individuals, while a medium textured bristle brush was used by overweight (62.3%) and obese (54.2%) participants. Majority of the underweight (64.3%), overweight (48.1%), and 45.8% of obese individuals had meals thrice a day, while healthy (62.3%) individuals had meals twice a day.

**Conclusion:**

This study further intensified the contributing role of having an excessive dietary intake and sugar consumption in causing obesity and dental caries. Findings from the current study, identify a statistically significant relationship that exists between BMI levels with oral hygiene, eating habits and the physical activity.

## Background

Dental students are future dentists who are responsible for providing preventive strategies, diagnosis, and treatment of dental problems to the community. Hence, they need to be well equipped with the knowledge and skills required by the field of dentistry to play their role effectively. In the modern era, the most challenging community health issue is the growing number of overweight and obese individuals [1]. Dental caries is consistently ranked as one of the world's most prevalent non-communicable diseases [2].

As stated by WHO, in 2016, almost 2 billion adults were overweight, with 40% women and 39% men, while 11% men and 15% women were found obese globally. There had been a noticeable increase in the number of obese and overweight individuals since the last four decades [3]. Irrespective of the genetic predisposition to obesity, the recent rapid changes within the environment along with readily available “high fat food” and with reduction in the level of physical activity have allowed increased obesity not only in developed countries but also in developing countries worldwide [4]. Furthermore it has also been identified that individuals having BMI within normal range generally have a fast metabolism as compared to obese who generally have lower resting metabolic rate, which can be attributed to high muscle mass that leads to higher resting metabolic rate [5]. Obese individuals’ body mass is made up of both fat and fat-free mass, and fat mass does not contribute considerably to metabolism [5] hence their metabolic rate is low.

Another important contributing factor was physical inactivity, which along with unhealthy dietary habits, has been linked with an increased incidence of obesity, osteoporosis and many other chronic degenerative and psychological diseases such as anxiety and depression [6, 7]. It has been reported that university students generally face difficulty in adopting a healthy lifestyle due to lack of physical and sporting activities coupled with improper nutrition [7] due to their hectic work schedules and increased study hours. As a result, students ignore healthier food options, regular physical activity, and oral hygiene maintenance in favour of convenience foods high in sugar and extra calories [8, 9]. A healthy and nutritious diet is one that provides appropriate amounts of proteins, carbohydrates, and fats and includes all the beneficial nutrients like vitamins, minerals, essential amino acids, and fatty acids plus dietary fibre [10].

Dental caries is a microbial infection that causes acidic dissolution and destruction of dental hard tissues [2]. The excessive consumption of free sugars has been linked with the development of many chronic conditions like obesity, diabetes, an increased risk of heart disease as well as dental caries [11]. It can be interpreted that an unhealthy diet is a risk factor for both dental caries and excess body weight [12]. Other than sugar, there are several other factors that contribute to caries development, including poor oral hygiene, cariogenic bacteria, and saliva volume [13]. Dental caries causes sensitivity and pulpal pain, which can lead to decline in the academic performance of dental students, loss of teeth, aesthetics problems, and lack of confidence [14, 15]. Body mass index (BMI) is a measurement of body fat based on adult men's and women's height and weight. BMI can be subdivided into four categories: underweight, normal weight, overweight, and obese [16]. Individuals having BMI in the range of 15 to 19.9, would be considered underweight, BMI from 20 to 24.9 would be recognised as normal weight, BMI was 25 to 29.9 will be regarded as overweight, while BMI 30 to 35 or more would be referred as obese [17].

Few research conducted in the past have focused on assessing the physical health of dental students. Research by Magliocca et al. identified that dental students and hygienists had insufficient knowledge regarding obesity [18]. Similarly, Awan et al. also identified that most of the study participants were unable to describe and identify the factors involved in obesity and the possible consequences associated with it [19]. Hence, dental students should not only have adequate knowledge regarding the oral hygiene practices, dietary counselling, and skill-based proficiencies but also practice healthy food consumption and oral hygiene habits themselves. Additionally, also must have sufficient knowledge regarding the physical inactivity and the possible medical risks associated with it [20]. So that they can educate their patients as well as be a positive role models for them. Hence, the biggest challenge in dental education is to prepare future dentists who can provide the health care to the patients, deal with the oral care crisis, and be competent enough to meet the global standards of excellence in dentistry [21]. The aim of the present study was to evaluate the association between BMI, physical activity, dietary habits, and oral hygiene practices of dental students studying at dental college of Pakistan.

### Method

This multi centric cross-sectional study was conducted at dental college from January to May 2021 in Pakistan. Three hundred and eighty-six study participants, including males and females, were enrolled as undergraduate dentistry students in the study. Convenience sampling technique was used for collection of data. Students who signed a written consent form and provided complete details were included in this study, while students who refused to provide permission for research were not included. It was made abundantly clear that participation in this research was entirely voluntary.

### Characterization of study sample

This study was carried out in accordance with the Helsinki ethical guidelines. Questionnaire used to gather data was modified from a recent study by Jouhar et al. [22]. The Cronbach’s alpha value for the questionnaire was found to be 0.72.

Current study questionnaire was sub-divided into three sections. The first part was comprised of demographic information where age, gender, height, weight, and year of study were inquired. Using the height and weight, BMI for each participant was calculated, and students were further subdivided as underweight, healthy (normal), overweight, and obese. The second section was comprised of eight questions related to the oral hygiene habits of the students. This included frequency of brushing teeth, whether it was done once, twice or thrice daily, type of toothbrush, manual or electric toothbrush (power operated), type of toothpaste used whether it was fluoridated, non-fluoridated, desensitizing, smoking habits and usage of interdental cleaning aids such as mouthwash, floss, and miswak. Miswak was added as Pakistan being a Muslim country and many participants would perform cleaning of teeth for religious reasons, using miswak. Brushing techniques using a manual toothbrush were also asked, which included horizontal, modified bass, modified stillman, Leonard’s method (vertical motion) and scrub method.

The third section involved seven questions related to diet and physical activity, which included frequency of meals taken in a day, if they were taken once, twice, thrice, or more than 3 times, and type of snack taken, which included chips, popcorn, biscuits, chocolates, and vegetables. The types of drinks consumed daily, comprised of tea, coffee, lemon juice, soft drinks, and orange juice. Green tea was also added as it is frequently used in Pakistan and has an inverse relationship with BMI. Alcohol was not included as its use is prohibited in Pakistan. Amount of workout done, if it was less than 30 min, more than 30 min or not done at all. Time spent watching TV and using the social media was also inquired, if it was used for 30 min, one hour, more than one hour, or not used at all.

### Statistical analysis

Using SPSS Program, Version 23.0, all the parameters were statistically studied at the subject level (SPSS Inc., Chicago, IL, USA). For condensing the raw data, frequency and percentage were determined as summary measures. To assess the relationship between two categorical variables, Chi-square testing was done, while linear regression was used to assess association of BMI and putative confounders. Statistical significance was defined as a measured of p value < 0.05.

## Results

From a total of 386, there were 300 females and 86 males. Majority of the participants were 20–23 years old (54.9%), while 174 were 18–20 years old (45.1%). From 1st year 166 students (43%), 150 students from 2nd year (38.8%), 56 (14.5%) from 3^rd^ year while 14 students (3.6%) from the final year participated. (Table 1).

**Table 1:** Demographic details of participants

Variable	n	%
*Age*		
18–20 years 20–23 years	174 212	45.1 54.9
*Gender*		
Females Males	300 86	77.7 22.2
*Year of education*		
1st year 2nd year 3rd year 4th year	166 150 56 14	43 38.8 14.5 3.6

Table 2 depicted association of oral hygiene habits with the BMI of participants. Regarding brushing teeth, 57% of underweight individuals brushed teeth once daily, 69.8% healthy, 79.2% overweight and 47.9% of obese participants brushed twice daily. Use of aids like floss, mouthwash, or miswak displayed statistically significant association amongst the overweight participants. Horizontal brushing technique was used by 50% of the underweight participants, followed by the scrub technique by the rest of the participants. Manual brushing with fluoridated toothpaste was performed by the majority of individuals. A soft bristled brush was preferred by underweight (42.9%) and healthy (66%) individuals, while a medium textured bristle brush was preferred by overweight (62.3%) and obese (54.2%) individuals. Most of the participants only visited the dentist when required. (Table 2).

**Table 2:** Oral hygiene habits association with BMI of the participants

Variable	Underweight	Healthy	Overweight	Obese	P value
*Tooth brushing*					
Once daily	16 (57.1%)	28(26.4%)	30(19.5%)	42(43.8%)	< 0.001**
Twice daily	10 (35.7%)	74(69.8%)	122(79.2%)	46(47.9%)	
Thrice daily	2 (7.1%)	4 (3.8%)	2(1.3%)	8(8.3%)	
*Interdental cleaning aids*					
Miswak	0	6 (5.7%)	28(18.2%)	8(8.3%)	0.004*
Floss	8(28.6%)	32(30.2%)	40(26%)	28(29.2%)	
Mouthwash	12(42.9%)	58(54.7%)	60(39%)	48(50%)	
*Brushing techniques*					
Horizontal	14(50%)	26(24.5%)	22(14.3%)	40(41.7%)	< 0.001**
Modified Bass	0	0	12(7.8%)	6(6.3%)	
Modified Stillman	0	4(3.8%)	0	0	
Scrub	10(35.7%)	52(49.1%)	80(51.9%)	42(43.8%)	
vertical	4(14.3%)	24(22.6%)	40(26%)	8(8.3%)	
*Type of toothbrush*					
Manual	24 (85.7%)	96 (90.6%)	148 (96.1%)	82 (85.4%)	0.022
Powered	4 (14.3%)	10(9.4%)	6 (3.9%)	14 (14.6%)	
*Type of toothpaste*					
Desensitizing	2(7.1%)	0	4 (2.6%)	2(2.1%)	0.038
Fluoridated	22(78.6%)	76(71.7%)	108(70.1%)	68(70.8%)	
Non fluoridated	2(7.1%)	0 (0)	4(2.6%)	0	
Don’t know	2(7.1%)	28(26.4%)	36(23.4%)	26(27.1%)	
*Texture of toothbrush bristle*					
Extra soft	0	2 (1.9%)	2(1.3%)	10(10.4%)	< 0.001**
Soft	12(42.9%)	70(66%)	56 (36.4%)	32(33.3%)	
Medium	16(57.1%)	30 (28.3%)	96(62.3%)	52(54.2%)	
Hard	0	4 (3.8%)	0	2(2.1%)	
*Frequency of mouthwash*					
After every meal	0	2(1.9%)	2(1.3%)	8(8.3%)	0.004*
None	10(35.7%)	44(41.5%)	70(45.5%)	46(47.9%)	
Once a day	14(50%)	32(30.2%)	54(35.1%)	34(35.4%)	
Twice a day	4(14.3%)	28(26.4%)	28(18.2%)	8(8.3%)	
*Frequency of visit to dentist*					
Every 3 months	2(7.1%)	4 (3.8%)	2(1.3%)	8(8.3%)	< 0.001**
After 6 months	0	10(9.4%)	4(2.6%)	2(2.1%)	
More than 6 months	0	0	8(5.2%)	4(4.2%)	
None	18(64.3%)	20(18.9%)	42(27.3%)	26(27.1%)	
Only when required	8(28.6%)	72(67.9%)	98(63.6%)	56(58.3%)	

Chi squared test

*P value less than 0.05 was considered significant

**P value less than 0.01 was considered significant

Table 3 showed the association of eating habits with the BMI of the participants. Regarding the frequency of meals taken in a day, healthy (62.3%) participants consumed meals twice daily, whereas others consumed 3 meals in a day. Consumption of chips showed statistically significant association in all groups, followed by chocolates and biscuits. Frequency of snacks intake was once daily, which demonstrated statistically significant association with all BMI categories. Tea consumption showed statistically significant association in all BMI groups. The frequency of physical activity was less than 30 min for all the participants, while screen time of more than one hour amongst all social media and TV categories showed statistically significant association in all categories. (Table 3).

**Table 3:** Association of eating habits with BMI of study participants

Variable	Underweight	healthy	Overweight	Obese	P value
*Frequency of meal in a day*					
One meal	0	4(3.8%)	2(1.3%)	4(4.2%)	0.004*
2 meals	8(28.6%)	66(62.3%)	64(41.6%)	44(45.8%)	
3 meals	18(64.3%)	28(26.4%)	74(48.1%)	44(45.8%)	
More than 3 meals	2(7.1%)	8(7.5%)	14(9.1%)	4(4.2%)	
*Snacks consumed between meals*					
Biscuits	6(21.4%)	38(35.8%)	18(11.7%)	28(29.2%)	0.00
Chips	14(50%)	42(39.6%)	72(46.8%)	44(45.8%)	
Chocolates	6(21.4%)	6(5.7%)	38(24.7%)	10(10.4%)	
Pop corn	0	10(9.4%)	10(6.5%)	6(6.3%)	
Vegetables	2(7.1%)	10(9.4%)	16(10.4%)	8(8.3%)	
*Frequency of snacks consumption*					
Once	14 (50%)	52 (49.1%)	84 (54.5%)	46 (47.9%)	0.317
Twice	6 (21.4%)	42 (39.6%)	48 (31.2%)	36 (37.5%)	
Thrice	2 (7.1%)	6 (5.7%)	10 (6.5%)	6 (6.3%)	
More than thrice	6 (21.4%)	6 (5.7%)	12 (7.8%)	8 (8.3%)	
*Type of drink consumed*					
Coffee	0	2(1.9%)	4(2.6%)	10(10.4%)	< 0.001**
Green tea	10(35.7%)	22(20.8%)	16(10.4%)	16(16.7%)	
Lemon juice	0	0	6(3.9%)	0	
Orange juice	0	6(5.7%)	8(5.2%)	2(2.1%)	
Soft drink	2(7.1%)	14(13.2%)	22(14.3%)	2(2.1%)	
Tea	16(57.1%)	62(58.5%)	98(63.6%)	66(68.8%)	
*Frequency of consuming drinks*					
Once a day	12(42,9%)	20(18.9%)	74(48.1%)	38(39.6%)	< 0.001**
Twice	10(35.7%)	56(52.8%)	46(29.9%)	46(47.9%)	
Thrice	0	10(9.4%)	6(3.9%)	2(2.1%)	
More than thrice	6(21.4%)	8(7.5%)	18(11.7%)	10(10.4%)	
*Frequency of workout*					
Less than 30 min	8 (28.6%)	50 (47.2%)	72 (46.8%)	34 (35.4%)	0.106
More than 30 min	8 (28.6%)	28 (26.4%)	30 (19.5%)	32 (33.3%)	
None	12 (42.9%)	28 (26.4%)	52 (33.8%)	30 (31.3%)	
*Frequency of watching TV & Social media*					
30 min	4 (14.3%)	2 (1.9%)	26(16.9%)	16(16.7%)	< 0.001**
1 h	4 (14.3%)	46(43.4%)	46(29.9%)	16 (16.7%)	
More than 1 h	18 (64.3%)	58(54.7%)	82(53.2%)	64(66.75%)	
None	2 (7.1%)	0	0	0	

Chi squared test

*P value less than 0.05 was considered significant

**P value less than 0.01 was considered significant

Table 4 determined the relationship between BMI and various testing variables performed using linear regression analysis. Association of BMI and frequency of drink intake can be clarified by B = − 0.109, 95% CI = − 0.194 to − 0.024, β = − 0.128, p = 0.012; BMI and tooth brushing by B = − 0.286, 95% CI = − 0.412 to − 0.160, β =− 0.223, p < 0.001. BMI when compared with mouthwash by B =− 0.178, 95% CI = − 0.289 to -0.068, β = − 0.160, p = 0.002. BMI association with social media use was explained by B = − 0.190 95% CI = − 0.349 to -0.030, β = − 0.120, p = 0.020). Statistically significant difference (p < 0.01) was noted when the variable such as frequency of drinks consumed, toothbrush type used, social media, smoking, and use of mouthwash were evaluated as confounders with the BMI in the current study. (Table 4).

**Table 4:** Association of BMI with the testing variables

Model	Unstandardized Coefficients		Standardized Coefficients	t	Sig	95.0% Confidence Interval for B	
(BMI vs Parameters)	B	Std. Error	Beta			Lower Bound	Upper Bound
Frequency of meals	0.001	0.07	0	0.008	0.994	− 0.133	0.134
Snacks taken between meals	0.019	0.04	0.026	0.503	0.615	− 0.056	0.095
Kind of drinks consumed	0.013	0.03	0.026	0.499	0.618	− 0.039	0.066
Frequency of consuming drinks	− 0.109	0.04	− 0.128	− 2.53	0.012	− 0.194	− 0.024
Consumption of snacks	− 0.027	0.05	− 0.028	− 0.54	0.59	− 0.125	0.071
Frequency of workout during the week	− 0.01	0.05	− 0.01	− 0.186	0.852	− 0.114	0.094
Frequency of physical Activity on weekend	0.042	0.06	0.037	0.715	0.475	− 0.073	0.156
Frequent of watching TV and using social media	− 0.19	0.08	− 0.12	− 2.338	0.02	− 0.349	− 0.03
Frequency of brushing teeth	0.008	0.09	0.005	0.089	0.929	− 0.162	0.178
Aids used for brushing	− 0.021	0.05	− 0.021	− 0.411	0.681	− 0.122	0.08
Type of brushing technique used	− 0.041	0.03	− 0.071	− 1.382	0.168	− 0.1	0.017
Smoking	0.548	0.21	0.133	2.615	0.009	0.136	0.96
Type of toothpaste used	− 0.078	0.08	− 0.048	− 0.936	0.35	− 0.241	0.086
Type of Toothbrush used	− 0.286	0.06	− 0.223	− 4.473	< 0.001	− 0.412	− 0.16
Frequency of using,outhwash	− 0.178	0.06	− 0.16	− 3.177	0.002	− 0.289	− 0.068
Frequency of visit to dentist	− 0.011	0.04	− 0.013	− 0.247	0.805	− 0.098	0.076

*B* B coefficient, *SE* standard error, *Wald* Wald chi-square test, *p* p-value, *OR* odds ratio, *CI* confidence interval

Significance taken at p < 0.05

Simple linear regression analysis, using testing variables as dependent variables, while BMI was used as independent variable

Table 5 identified relationship between frequency and type of drinks consumed. It can be seen that tea was mostly consumed once by 88 (60.3%) participants and twice in a day by 112 (70.9%), while 32 (50%) participants consumed more than 3 times in a day. (Table 5).

**Table 5:** Association of drinks intake with frequency

Frequency	Coffee	Green Tea	Lemon Juice	Orange Juice	Soft Drink	Tea	P-value
Once/day	10 (6.8%)	24 (16.4%)	6 (4.1%)	4 (2.7%)	14 (9.6%)	88(60.3%)	
Twice/day	6 (3.8%)	18 (11.4%)	0	10 (6.3%)	12 (7.6%)	112 (70.9%)	
Thrice/day	0	4 (22.4%)	0	0	4 (22.2%)	10 (55.6%)	0.001
More than 3 /day	0	20 (31.3%)	0	2 (3.1%)	10 (15.6%)	32(50%)	

Chi squared test

P value less than 0.05 was considered significant

## Discussion

Food intake and physical activity have a direct impact on the body mass index along with lifestyle habits, such as the frequency of sugary food intake. These factors tend to affect the general as well as the oral health of individuals [23]. Obesity and dental caries have many different aetiologies but have a mutual contributing factor which is diet high in sugar [24]. Dental caries has been recognized as the one of the most widespread disease worldwide [25] and causes significant oral health problems. It affects about 60% to 90% of school children*,* and most of the adults [26]. Caries has a complex aetiology and comprises of various contributory factors including diet, oral hygiene, saliva, and oral microflora that affects its development and progression [27]. The literature displays contradictory evidence regarding the association between a patient’s BMI and the status of their oral wellbeing and hygiene [28]. Some previously conducted research showed a substantial association between overweight/obesity and high caries incidence [29, 30] whereas some studies claimed that there was no relationship whatsoever between BMI and dental caries [31, 32]. However, obesity and dental caries have common risk factors, and hence dental healthcare experts should employ a professional approach to treat it.

In the current study, the association of BMI with the oral hygiene habits for the dental students was assessed. Statistically significant association was seen between the majority of the participants having different BMI who brushed their teeth twice daily. These findings are in contrast with the study led by Chang et al., who identified that individuals who brushed their teeth twice daily had lower BMI [33]. These findings can be attributed to the fact that in the current study, dental students were evaluated, they had better knowledge regarding the importance of brushing teeth twice daily. Statistically significant association was seen between the using manual toothbrush (P = 0.022) and the use of fluoridated toothpaste (P = 0.038) in different BMI groups. These findings are in agreement with the previously conducted research [22]. Similarly, research conducted in Japan, identified positive association between obesity and lower frequency of brushing habits [34]. Similarly, research by Park et al. also identified positive association between obesity and time of brushing teeth and use of mouthwash [35]. This is because poor oral hygiene aside from causing an inflammatory response within the oral cavity, also causes increase in the levels of C reactive protein, which has been linked with the obesity [35]. Brushing with a fluoridated toothpaste helps in removal of bacterial biofilm present on surface of teeth, which is strongly linked with aetiology of several oral diseases like periodontal diseases, dental caries and pulpal diseases [36]. Hence the importance of maintaining good oral hygiene must be emphasised as being overweight or obese has been strongly associated with periodontal diseases. [36] Moreover when we assessed the knowledge of final year students, majority of the students brushed using fluoridated toothpaste with a manual toothbrush. Similar findings have been reported by Tadin et al. where majority of the students brushed using a manual brush [37]. As expected, final year dental students had adequate knowledge regarding importance of brushing using a fluoridated tooth paste.

Tooth brushing is a mechanical mean of plaque removal owing to its effectiveness, accessibility, as well as cost effectiveness. Various brushing techniques have been introduced [38]. In the current study, horizontal brushing technique was mostly preferred by the participants. Likewise, in the current study, brushing technique was positive correlated with different BMI groups. Poor oral hygiene has a direct association with inflammation within the oral cavity and obesity [39]. Additionally, the link between tooth brushing and obesity is thought to be due to leptin-linked pathways that controls the balance between energy and appetite. As a result, regular and proper tooth brushing technique can aid in suppressing hunger and lowers the risk of obesity [35].

In this study, a statistically significant association was seen regarding using mouthwash as the most common aid (P = 0.004) with BMI. These findings are in agreement with the study conducted by Jouhar et al. [22]. Previously conducted research by Damyanov et al. and Melo et al. have reported positive influence of adequate oral hygiene practices on dental wellbeing. [40, 41].

Dietary habits are important contributing factor for weight gain and can also cause dental caries. Changes in the dietary pattern due to frequent consumption of fast food, sugary drinks, and products containing refined sugar have been identified as common risk factors for obesity and dental caries [42]. In the current study, most of the obese and overweight participants consumed meals thrice a day. These findings are in contrast with the study carried out by Aljuraiban et al. who identified that frequent meals had statistically significant association with low BMI [42]. It has been identified that reducing the frequency of meals per day may have an adverse effect on appetite control [43]. There is evidence that hormonal and nutritional signals can suppress the appetite, when combined with an increase in the frequency of meals [44]. This results in decreased energy levels and delayed stomach emptying, which reduces the feeling of hunger [45].

Snacks can be best described as small meals or drinks frequently taken in between the meals. However, this also depends on the frequency and quantity of food or drink consumed between meals [46]. Also repeated snacking in between meals has been linked with obesity and other chronic diseases. It was noted in a study by Alswat et al. that the incidence of caries was more prevalent in younger population with high BMI, who consumed drinks high in sugar and had a sedentary lifestyle [47]. This is because obese and overweight individuals spent more time online using social media, hence it was combination of sedentary lifestyle and frequent snacking that contributed towards the weight gain. Moreover, final year students when compared to other students, consumed snacks once a day. These findings are in line with the study conducted by Aljefree et al. where less than half of the surveyed dental students had snacks once daily, hence this reduced the incidence of gaining weight [48].

In the current study, chips were often consumed in between meals by most of the participants, followed by biscuits and chocolates. Very few participants consumed popcorn and vegetables for snacking. It was also seen that the majority of the students, whether underweight, healthy or overweight/obese, consumed snacks at least once a day. Dietary habits have a significant role in both the obesity and dental caries epidemics. Final year students also snacked on chips followed by biscuits, similar findings have been reported by Aljefree et al. where chips were the most common snacking item, [46].

In the current study, few students from all BMI groups performed physical activity for 30 min, while most of the participants performed physical activity for less than half an hour. These students spent substantial amount of time watching TV and using social media. Hence some led a sedentary lifestyle with no physical activity at all. A study done by Finlayson et al. also proved that there was a significant decrease in all types of physical activity amongst the students as they began university [7]. This shift to sedentary lifestyles, as well as an increase in fast food consumption along with lack of adequate sleep due to the burden of studies, all contributed towards the weight gain [49, 50]. Alm et al. identified a statistically significant relationship between dental caries and unhealthy dietary habits that ultimately leads to obesity [51].

### Practical implications

Dental caries and obesity share common contributing and modifiable factors such as diet and lifestyle, including transition to reduced physical activity and consumption of highly processed and sugar loaded foods. Obesity can be regarded as a predictor of dental caries, and obese people need more frequent dental examinations, dental care, and oral health education. Dental students should recognize their role in creating general awareness in the community about the prevention of disease and the promotion of health and become positive role models for the community.

### Limitations

Few limitations from the current study have been identified, the present study followed a cross-sectional design, restricting the results’ significance for determining causal associations. [52] Secondly although this research was multi centric, however due to COVID-19 pandemic response rate received was quite less. In the current study, only dental students were included, for future research, participants from other relevant medical fields or others can also be involved. The participants’ oral health status was not assessed, which should be done for future research to determine a direct relationship between oral hygiene and BMI. The upcoming researchers can produce longitudinal observational surveys that could help to verify the present results and clarify the association between BMI and dental caries, obesity, and nutritional status.

## Conclusion

This study further intensified the contributing role of having an excessive dietary intake and sugar consumption in causing obesity and dental caries. Findings from the current study, identify a statistically significant relationship that exists between BMI levels with oral hygiene, eating habits and the physical activity.

## Data Availability

The data for this study was obtained dental institute of Pakistan, which have restricted the authors for sharing their data hence data is not publicly available. The dataset used for the current study are available from the corresponding author upon reasonable request.
